# Deletion of the Sodium-Dependent Glutamate Transporter GLT-1 in Maturing Oligodendrocytes Attenuates Myelination of Callosal Axons During a Postnatal Phase of Central Nervous System Development

**DOI:** 10.3389/fncel.2022.905299

**Published:** 2022-06-03

**Authors:** Elizabeth J. Thomason, Edna Suárez-Pozos, Fatemah S. Afshari, Paul A. Rosenberg, Jeffrey L. Dupree, Babette Fuss

**Affiliations:** ^1^Department of Anatomy and Neurobiology, Virginia Commonwealth University School of Medicine, Richmond, VA, United States; ^2^Department of Neurology and the F.M. Kirby Neurobiology Center, Boston Children’s Hospital, Boston, MA, United States

**Keywords:** glutamate transport, oligodendrocyte, myelination, development, conditional and inducible knockout

## Abstract

The sodium-dependent glutamate transporter GLT-1 (EAAT2, SLC1A2) has been well-described as an important regulator of extracellular glutamate homeostasis in the central nervous system (CNS), a function that is performed mainly through its presence on astrocytes. There is, however, increasing evidence for the expression of GLT-1 in CNS cells other than astrocytes and in functional roles that are mediated by mechanisms downstream of glutamate uptake. In this context, GLT-1 expression has been reported for both neurons and oligodendrocytes (OLGs), and neuronal presynaptic presence of GLT-1 has been implicated in the regulation of glutamate uptake, gene expression, and mitochondrial function. Much less is currently known about the functional roles of GLT-1 expressed by OLGs. The data presented here provide first evidence that GLT-1 expressed by maturing OLGs contributes to the modulation of developmental myelination in the CNS. More specifically, using inducible and conditional knockout mice in which GLT-1 was deleted in maturing OLGs during a peak period of myelination (between 2 and 4 weeks of age) revealed hypomyelinated characteristics in the corpus callosum of preferentially male mice. These characteristics included reduced percentages of smaller diameter myelinated axons and reduced myelin thickness. Interestingly, this myelination phenotype was not found to be associated with major changes in myelin gene expression. Taken together, the data presented here demonstrate that GLT-1 expressed by maturing OLGs is involved in the modulation of the morphological aspects associated with CNS myelination in at least the corpus callosum and during a developmental window that appears of particular vulnerability in males compared to females.

## Introduction

The sodium-dependent glutamate transporter GLT-1 (EAAT2, SLC1A2) has been well-characterized for its role in regulating extracellular glutamate homeostasis in the central nervous system (CNS), a function that is performed mainly through its presence on astrocytes (Rothstein et al., 1996; Tanaka et al., 1997; Danbolt, 2001; Petr et al., 2015; Rose et al., 2018). In general, glutamate uptake by sodium-dependent glutamate transporters is driven by co-transport of sodium ions and protons, as well as counter-transport of potassium ions (Rose et al., 2018). The complexity of this transport mechanism allows active transport of glutamate into the cell against a steep concentration gradient. At the same time, it enables functional consequences that are beyond the regulation of glutamate homeostasis and have in astrocytes been shown to include alterations in brain energetics and intracellular signaling (Chen et al., 2006; Martinez-Lozada et al., 2011; Maria Lopez-Colome et al., 2012; Flores-Mendez et al., 2013; Robinson and Jackson, 2016; Robinson et al., 2020). Such multifaceted roles of sodium-dependent glutamate transporters are further broadened by their expression in CNS cells other than astrocytes. In the case of GLT-1, its neuronal presence at presynaptic sites has been functionally implicated not only in glutamate uptake but also in the regulation of gene expression and mitochondrial function (Petr et al., 2015; Rimmele and Rosenberg, 2016; Laprairie et al., 2019; McNair et al., 2019, 2020; Zhou et al., 2019; Rimmele et al., 2021). GLT-1 expression and GLT-1 mediated glutamate uptake have also been reported for the myelinating cells of the CNS, namely oligodendrocytes (OLGs) (Domercq and Matute, 1999; Regan et al., 2007; Arranz et al., 2008; DeSilva et al., 2009; Kukley et al., 2010; Lee A. et al., 2012; Martinez-Lozada et al., 2014; Suarez-Pozos et al., 2020). However, the extent to which OLG expressed GLT-1 may exert functional outcomes *via* mechanisms downstream of its transporter activation remains largely unknown.

First evidence for potential roles of OLG expressed GLT-1 beyond glutamate uptake were revealed by our previous studies in which activation of sodium-dependent glutamate transporters and particularly GLT-1 was shown to enhance the morphological aspects of OLG maturation (Martinez-Lozada et al., 2014; Spencer et al., 2020; Suarez-Pozos et al., 2020; Thomason et al., 2020). More specifically, glutamate uptake was found to be associated with a reversal of sodium-calcium exchange leading to intracellular calcium transients and alterations in actin cytoskeletal organization (Martinez-Lozada et al., 2014; Spencer et al., 2020; Suarez-Pozos et al., 2020) that likely drive process outgrowth and branching (Martinez-Lozada et al., 2014; Thomason et al., 2020) and, thus, morphological alterations thought to be crucial for effective initiation of CNS myelination (Zuchero et al., 2015; Seixas et al., 2019). Based on these data, OLG expressed GLT-1 emerges as a potential modulator of CNS myelination. In order to better assess such a functional role of GLT-1, we generated inducible and conditional knockout mice in which GLT-1 can be deleted from maturing OLGs during the peak period of myelination. Using this approach, our data shown here revealed hypomyelinated features in the male corpus collosum that were not found associated with significant changes in myelin gene expression. Thus, our findings demonstrate that OLG expressed GLT-1 can modulate developmental CNS myelination, at least in the male corpus callosum, by controlling a morphology regulatory program that functions largely independent from the well-described program directing myelin gene expression.

## Materials and Methods

### Reagents and Antibodies

Unless otherwise stated, all reagents were purchased from Sigma-Aldrich or Thermo Fisher Scientific. Details to antibodies are listed below and PCR primers are shown in Table 1.

**TABLE 1:** List of primer sequences used for RT-qPCR analysis.

	Forward primer (5’–3’)	Reverse primer (5′–3’)
**Mouse genes**		
*Mbp*	GTGACACCTCGTACACC CCCTCCA	GCTAAATCTGCTGA GGGACAGGCCT
*Plp1*	CCACACTAGTTTCCCT GCTCACCT	GGTGCCTCGGC CCATGAGTT
*Mog*	ATCTGCTACAACTGG CTGCAC	GGGAAATCCCCAAG GACCTGC
**Mouse reference genes**		
*Ppia*	GGAGACGAACCTGTA GGACG	GATGCTCTTTCCTC CTGTGC
*Pgk1*	ATGCAAAGACTGGCCA AGCTAC	AGCCACAGCCTCAG CATATTTC
*Rpl13a*	GCGCCTCAAGGTGT TGGATG	CGCCCCAGGTAAGC AAACTTTC

*Mbp, myelin basic protein; Mog, myelin oligodendrocyte glycoprotein; Pgk1, phosphoglycerate kinase 1; Plp1, proteolipid protein; Ppia, peptidylprolyl isomerase A (cyclophilin A); Rpl13a, ribosomal protein L13a.*

For flow cytometry, Fc receptors were blocked using anti-CD16/CD32 antibodies (rat IgG2a, 1:50, Thermo Fisher Scientific Cat# 14-0161-86, RRID:AB_467135). The following antibodies were used to determine the percentage of GLT-1-positive maturing OLGs: O4 (phycoerythrin-conjugated, mouse IgM, 10 μl/million cells, R&D Systems Cat# FAB1326P, RRID:AB_664169), isotype control to O4 (phycoerythrin-conjugated, mouse IgM, 10 μl/million cells, R&D Systems Cat# IC015P, RRID:AB_2885000), anti-EAAT2/GLT-1 (rabbit polyclonal, 1:50, Novus Cat# NBP1-20136, RRID:AB_1641916), isotype control to anti-EAAT2/GLT-1 (rabbit IgG polyclonal, 1:50, Novus Cat# NBP2-24891, RRID: AB_2811130), and Alexa 633-conjugated anti-rabbit secondary antibodies (goat anti-rabbit IgG, Molecular Probes Cat# A-21071, RRID:AB_141419).

For immunocytochemistry, anti-APC (CC-1; mouse IgG_2*b*_, 1:100, Millipore Cat# OP80, RRID:AB_2057371) antibodies in combination with goat anti-mouse IgG_2*b*_, AlexaFluor 568 conjugated secondary antibodies (1:00, Thermo Fisher Scientific Cat# A-21144, RRID:AB_2535780). YFP was detected using anti-GFP antibodies (rabbit polyclonal IgG, 1:100, Millipore Cat# AB3080, RRID:AB_91337) in combination with Alexa 488-conjugated secondary antibodies (1:500, goat anti-rabbit IgG, Thermo Fisher Scientific Cat# A-11008, RRID:AB_143165).

### Animals

*Glt-1^flox^* (*Slc1a2^TM^* ^1^.^1^*^Pros^*; MGI: 5752263) mice were generated as previously described (Petr et al., 2015) and crossbred to *Plp1^CreERT^* (RRID:IMSR_JAX:005975) driver (Doerflinger et al., 2003) and *ROSA-EYFP* [*Gt(ROSA)26Sor^TM^* ^1^(*^EYFP)Cos^*; RRID:IMSR_JAX:006148] reporter (Srinivas et al., 2001) mice to obtain *Glt-1^flox/flox^*:*Plp1^CreERT^*^±^:*ROSA-EYFP* mice, here referred to as *Glt-1*^Plp1^*icKO* mice; animals were kept heterozygous for *Plp1^CreERT^* and on a C57BL/6J background. *Plp1^CreERT^*-negative littermates were used as controls and are referred to as *Wt*. Recombination was induced by intraperitoneal injection of 4-hydroxy-tamoxifen (4-HT; 1 mg; Sigma Aldrich Cat# H7904) at P14, P16, and P17, followed by analysis at 4 weeks of age; both *Glt-1 ^plp^*^1^*icKO* and *Wt* mice were treated with 4-HT. Genotypes for all animals were confirmed by genomic PCR using tail tissue; in the flow cytometry and RT-qPCR experiments 4-HT-induced recombination was confirmed by genomic PCR using spinal cord and brain tissue, respectively. Mice were housed in humidity- and temperature-controlled spaces, on a normal 12/12 h light/dark cycle, with free access to standard chow and water. All animal studies were approved by the Institutional Animal Care and Use Committees at Virginia Commonwealth University (IACUC #AM10229).

### Confocal and Electron Microscopy

For confocal imaging, 40 μm cryostat sections were prepared and immunstained in principle as previously described (Dupree et al., 1999; Benusa et al., 2017). Briefly, male and female *Glt-1 ^plp^*^1^*icKO* and control mice were anesthetized and transcardially perfused with 4% paraformaldehyde in 0.1 M Millonig’s phosphate buffer (Karlsson and Schultz, 1965), and brains were removed, post-fixed for 24 h in perfusion fixative, cryoprotected by immersion in 30% sucrose in PBS for 48 h, and then embedded and frozen in Tissue-Tek O.C.T. compound (Sakura Finetek Cat# 4583). Serial coronal sections were prepared using a Leica CM 1850 cryostat and stored at -80°C. Tissue sections were permeabilized for 10 min in ice-cold acetone and blocked for 1 h at room temperature using PBS containing 1% Triton X-100 and 5% cold water fish skin gelatin (Electron Microscopy Science cat# 25560). Primary antibodies were diluted in blocking solution, and sections were incubated for 48 h at 4°C followed by incubation with secondary antibodies for 90 min at room temperature. Nuclei were counterstained using Hoechst 33342 and sections were mounted using Vectashield Antifade Mounting Medium (Vector Laboratories Cat# H-1000-10). Images were collected using a Zeiss LSM 710 confocal laser scanning microscope located within VCU’s Microcopy Shared Resource. Confocal z-stacks, each spanning an optical distance of 15 μm, were collected at 1 μm intervals using a 40x oil-immersion objective with a numerical aperture of 1.3 and the following settings: a pinhole size of one Airy unit, a dimension of 1248×1248 pixels and 4 times line averaging. For image acquisition and the generation of maximum intensity projections ZEN imaging software (Carl Zeiss Microscopy, LLC, Thornwood, NY, United States; RRID:SCR_013672) was used. For electron microscopy, brain tissue was prepared as previously described (Dupree et al., 1998; Marcus et al., 2006; Forrest et al., 2009; Waggener et al., 2013). In brief, male and female *Glt-1 ^plp^*^1^*icKO* and control mice were deeply anesthetized, transcardially perfused and post-fixed for 2 weeks. Brains were harvested and 60 μm sagittal sections were prepared on a Leica VT1000S Vibratome. Sections were processed for standard EM analysis as previously described (Dupree et al., 1998). Ultrathin sections were imaged using a Jeol JEM-1400Plus equipped with a Gatan CCD camera and located within VCU’s Microcopy Shared Resource; to ensure comparable area representation within the corpus callosum, the following structures were used as landmarks: fornix, longitudinal lines, hippocampus, and cortex. 10–20 images obtained at 5000x magnification representing a minimum of 100 axons with diameters > 0.3 μm were assessed per animal (Mason et al., 2001); axon diameter, myelin thickness and *g*-ratio were determined manually using Fiji-ImageJ (Schindelin et al., 2012).

### Flow Cytometry

Male and female *Glt-1 ^plp^*^1^*icKO* and control mice were euthanized using intraperitoneal injection of an overdose of tribromomethanol (2–3 times 250 mg/Kg, Sigma Aldrich Cat# T48401) and brains were removed and coarsely chopped with microdissecting scissors. Single cell suspensions were prepared by enzymatic digestion at 37°C/CO_2_ for 30 min (1 ml per brain, StemPro Accutase, Thermo Fisher Scientific Cat# A1110501) followed by enzyme inactivation [addition of 2 ml 10% fetal bovine serum (FBS) in Hank’s Balanced Salt Solution (HBSS)], physical dissociation *via* trituration and filtration using 100 μm cell strainers. To purify cells from myelin debris, cells were resuspended in 40% Percoll (GE Healthcare Cat# 17-0891-02) in HBSS and centrifuged at 650 × *g* without brake for 25 min at room temperature. The myelin top layer was aspirated and cells were resuspended in Dulbecco’s Modified Eagle’s Medium (DMEM)/10% FBS also used as immuno-labeling buffer. 10^6^ cells per sample were used for immuno-labeling and analysis. Briefly, Fc receptors were blocked using CD16/CD32 antibodies (15 min on ice) followed by incubation with primary antibodies (or isotype control; 30 min on ice), fluorescently labeled secondary antibodies (30 min on ice), and 7-AAD Viability Staining Solution (5 μl/million cells, Thermo Fisher Scientific Cat# 00-6993-50). Cells were resuspended in flow cytometry buffer [0.1% bovine serum albumin (BSA) in phosphate buffered saline (PBS) containing 2 mM ethylenediaminetetraacetic acid (EDTA)] and run on an LSRFortessa-X20 flow cytometer (BD Biosciences). Settings were carefully determined empirically and exactly reproduced in each experiment. Gates were demarcated to count phycoerythrin and Alexa 633 double positive (7-AAD-negative) cells up to 10,000 events. FACSDIVA software (BD Biosciences, RRID:SCR_001456) was used for acquisition, and data were analyzed using FCS Express Flow Cytometry software (*DeNovo* Software, RRID:SCR_016431).

### RNA Isolation and RT-qPCR

RNA was purified from dissected corpus callosum tissue using a Qiagen RNeasy Micro kit (Qiagen Cat# 74004). RNA quality and concentrations were determined on a 2100 Bioanalyzer (Agilent) using an RNA 6000 Pico kits (Agilent Cat# 5067), and samples with an RNA integrity number above 7 were used for further analysis. cDNA synthesis was performed using a Sensiscript reverse transcription kit (Qiagen Cat# 205213) according to the manufacturer’s guidelines. RNA samples were normalized to the same approximate concentration, and the same amount of RNA was used for all conditions of an individual independent RT-qPCR experiment.

For all RT-qPCR experiments the Minimum Information for Publication of Quantitative Real-Time PCR *E*xperiments (MIQE) guidelines were followed (Bustin et al., 2009). Briefly, RT-qPCR primers (Table 1) were designed and *in silico* tested for specificity using the National Center for Biotechnology Information’s basic local alignment search tool (Primer-BLAST) (Ye et al., 2012). All primers were designed to amplify all known splice variants, and for all primer pairs melting curves were used to ensure specificity. cDNA reactions without reverse transcriptase were performed for all samples to ensure no-reverse-transcriptase quantitation cycle (C_*q*_) numbers of at least five cycles below the lowest C_*q*_ for any of the experimental samples. RT-qPCR reactions with three technical replicates per sample were performed on a CFX96 real-time PCR detection system (Bio-Rad) using the iTaq Universal SYBR Green Supermix (Bio-Rad Cat# 1725121). PCR conditions were set to 95°C for 3 min followed by 40 cycles of 95°C for 15 s, 58°C for 30 s, and 95°C for 10 s. Relative expression levels were determined using the ΔΔCT method relative to the geometric mean of the three reference genes (Livak and Schmittgen, 2001).

### Western Blot Analysis

For Western blot analysis, dissected mouse corpus callosum samples were homogenized in lysis buffer [10 μL per mg tissue; phosphate-buffered saline (PBS), 2 mM EDTA] containing 1x Halt protease and phosphatase inhibitor cocktail (Thermo Fisher Scientific Cat# 78430). Protein concentrations were determined using a BCA protein assay kit (Pierce/Thermo Fisher Scientific Cat# 23225). Equal amounts of denatured protein samples (60 μg) were separated by electrophoresis through 4–20% gradient sodium dodecyl sulfate (SDS)-polyacrylamide gels (Bio-Rad Cat# 4561094) and electroblotted onto Immobilon-P polyvinylidene difluoride (PVDF) membranes (MilliporeSigma Cat# IPVH00010). For normalization, membranes were incubated with a Revert 700 Total Protein Stain (LI-COR Biosciences Cat# 926-11011) and imaged on an Odyssey infrared imaging system. Subsequently, membranes were incubated in blocking buffer (LI-COR Biosciences Cat# 927-70001) for 1 h at room temperature followed by incubation with anti-proteolipid protein (PLP) rat hybridoma supernatants [clone AA3 (Yamamura et al., 1991); kindly provided by Dr. Wendy Macklin, University of Colorado] for 48 h at 4°C. Bound primary antibodies were detected using IRDye 680RD-conjugated secondary antibodies (LI-COR Biosciences Cat# 926-68076, RRID:AB_10956590). For quantification, membranes were imaged using an Odyssey infrared imaging system and analyzed using Image Studio (LI-COR Biosciences software, RRID:SCR_015795) and Empiria Studio software packages (LI-COR Biosciences software Cat# 9141-500E).

### Statistical Analysis

GraphPad Prism (GraphPad Software Inc., RRID:SCR_002798) was used for all statistical analyses. Prior sample size calculations were not performed. Data were assessed for normality using the Shapiro-Wilk normality test prior to analysis. Data compared with a set control value lacking variability were analyzed using the one-sample *t*-test (Skokal and Rohlf, 1995; Dalgaard, 2008). Data with two groups were analyzed by unpaired *t*-test (two-tailed), in case of pairs of *Glt-1 ^plp^*^1^*icKO* and control mice per litter a nested *t*-test was used, and comparisons with two parameters were analyzed for significance using two-way ANOVA with Tukey *post-hoc* testing. Frequency distributions were analyzed using Gompertz growth curves as a non-linear regression model, followed by an *F*-test to determine statistical significance.

## Results

**FIGURE 1:**
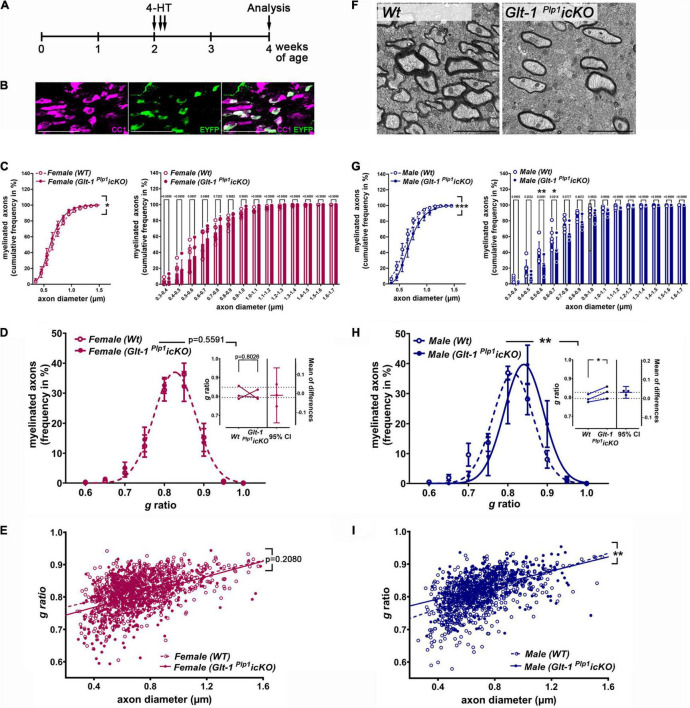
Conditional deletion of *Glt-1* from maturing OLGs attenuates CNS myelination in the corpus callosum of male mice. **(A)** Scheme depicting the timing of intraperitoneal injections of 4-hydroxytamoxifen (4-HT; P14,16,17) in *Glt-1 ^Plp^*^1^*icKO* and *Wt* (*Plp1^CreERT^*-negative littermate) mice and the developmental time-point of analysis (4 weeks of age). **(B)** Representative confocal image of the corpus callosum (midline); CC1 marks maturing OLGs (Fuss et al., 2000; Bin et al., 2016) and EYFP, derived from the ROSA reporter, marks cells in which Cre-mediated recombination has occurred successfully (Srinivas et al., 2001). Sale Bar: 50 μm. **(C,G)** Line (left) and bar (right) graphs depicting the cumulative frequency distribution of myelinated fibers in the corpus callosum of *Wt* (open circles) and *Glt-1 ^Plp^*^1^*icKO* (filled circles) female **(C)** and male **(G)** mice based on axon diameter; for the line graphs, the Gompertz growth curve was used as non-linear regression model (least squares regression) and extra sum-of-squares *F*-test was used to determine significant differences between regression curves; for the bar graphs, two-way ANOVA and Šidák multiple-comparisons test were used [*p* = 0.0307 (female) and *p* < 0.0001 (male) for overall difference between genotypes]. **(D,H)** Line graphs depicting the frequency distribution of myelinated fibers in the corpus callosum of *Wt* (open circles) and *Glt-1 ^Plp^*^1^*icKO* (filled circles) female **(D)** and male **(H)** mice based on *g*-ratio. Gaussian distribution was used as non-linear regression model (least squares regression); extra sum-of-squares *F*-test was used to determine significant differences between regression curves. The insets depict mean *g*-ratios per animal pairs of individual litters; paired *t*-test (between individual animals from the same litter; data are shown for 3 litter pairs) was used to determine significance. **(E,I)** Scatter plots depicting g-ratios vs. axon diameter for the corpus callosum of *Wt* (open circles) and *Glt-1^Plp^*^1^*icKO* (filled circles) female **(E)** and male **(I)** mice. The lines represent linear regression fits to pooled data from all animals for each genotype; an *F*-test was used to determine significant differences between the slopes of the regression curves. **(F)** Representative electron microscopic images of the corpus callosum of *Glt-1 ^Plp^*^1^*icKO* and *Wt* (*Plp1^CreERT^*-negative littermate) male mice injected with 4-HT. Scale bar: 2 μm. female *Glt-1 ^Plp^*^1^*icKO* (*n* = 5), female *Wt* (*n* = 6), male *Glt-1 ^Plp^*^1^*icKO* (*n* = 4), male *Wt* (*n* = 6). *p ≤ 0.05, **p ≤ 0.01, ***p ≤ 0.001.

In order to investigate developmental myelination upon deletion of *Glt-1* in maturing OLGs, *Glt-1 ^Plp^*^1^*icKO* and *Wt* (*Plp1^CreERT^*-negative littermate) mice were treated with 4-HT at postnatal days 14, 16, and 17, and myelination of the corpus callosum was investigated at 4 weeks of age (Figure 1A). The above-described timing was chosen since it coincides with the reported developmental expression of *Glt-1* in OLGs (Regan et al., 2007; DeSilva et al., 2009) and a period of active myelination in the corpus callosum (Caley and Maxwell, 1968; Foran and Peterson, 1992; Leone et al., 2003). In addition, while *PLP^CreERT^*-driven recombination has been reported to occur in cells other than OLGs (Kang et al., 2010), it has been established to be largely restricted to OLG lineage cells when induced around postnatal day 16 and beyond (Michalski et al., 2011). Consistently, when using the strategy outlined in Figure 1A and assessing successful recombination *via* EYFP expression derived from the ROSA-EYFP reporter, more than 95% of EYFP positive cells were also found to be positive for CC1, a marker for maturing OLGs (Fuss et al., 2000; Bin et al., 2016; Figure 1B). Few (less than 5%) EYFP positive cells were observed to be CC1-negative; based on previously published data, these cells likely represent OLG progenitor cells (Guo et al., 2009). At 4 weeks of age, recombination could be detected in approximately 50% of CC1 positive maturing OLGs, an effect level that is consistent with previously published studies demonstrating clear functional consequences (Traka et al., 2010; Koenning et al., 2012; Jeffries et al., 2016). Importantly, the inducible *Plp1^CreERT^* system has been used successfully by us (Forrest et al., 2009) and others (Doerflinger et al., 2003; Kang et al., 2010; Michalski et al., 2011) with no evidence of Cre-mediated toxicity.

Assessing myelination of the corpus callous at 4 weeks of age by ultrastructural analysis, revealed a slight shift toward smaller diameter axons in female *Glt-1 ^Plp^*^1^*icKO* mice (Figure 1C). However, pairwise comparisons per 0.1 μm axon diameter bins revealed no differences, suggesting a slight increase of myelination over a larger range of smaller diameter axons. In contrast to females, a significant shift toward larger diameter myelinated axons was observed in male *Glt-1 ^Plp^*^1^*icKO* mice (Figures 1F,G). Notably, this effect appears largely due to a decrease in myelination of axons within a narrow range of diameter, namely between 0.5 and 0.7 μm (bar graph in Figure 1G). No change in myelin thickness, as assessed by calculating *g*-ratios, was noted in female *Glt-1 ^Plp^*^1^*icKO* mice (Figure 1D). In contrast, in male *Glt-1 ^Plp^*^1^*icKO* mice an increase in *g*-ratios, reflecting thinner myelin sheaths, was noted (Figures 1G,H). In particular, despite variances between animals from different litters, the increase in *g*-ratio seen in male *Glt-1 ^Plp^*^1^*icKO* mice appears consistent when evaluating pairs of *Wt* and *Glt-1 ^Plp^*^1^*icKO* mice within individual litters (see inset in Figure 1H). Notably, these alterations in myelination were not found accompanied by obvious signs of widespread cell death or axonal pathology (Figure 1F). Furthermore, plotting *g*-ratios vs. axon diameter, revealed that the differences in *g*-ratios between *Glt-1 ^Plp^*^1^*icKO* and *Wt* (*Plp1^CreERT^*-negative littermate) male mice is most prominently seen for smaller diameter myelinated axons (Figure 1I). Thus, the increase in *g*-ratio (and reduction in myelin thickness) in the corpus callosum of *Glt-1 ^Plp^*^1^*icKO* male mice does not reflect an indirect effect due to the observed shift toward larger diameter myelinated axons; instead, it largely represents an additional characteristic of the hypomyelination phenotype that is seen in the male *Glt-1 ^Plp^*^1^*icKO* corpus callosum at 4 weeks of age and that appears to be particularly relevant to smaller diameter axons.

**FIGURE 2:**
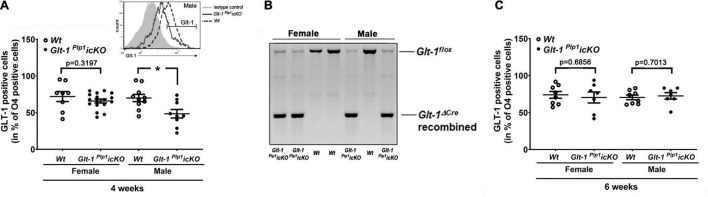
The percentage of GLT-1-positive maturing (O4-positive) OLGs is transiently reduced in male *Glt-1 ^Plp^*^1^*icKO* brains at 4 weeks of age. **(A,C)** Graphs depicting the percentage of maturing (O4-positive) OLGs with surface expression of GLT-1, as determined by flow cytometry, at 4 **(A)** and 6 **(C)** weeks of age. *Glt-1 ^Plp^*^1^*icKO* and *Wt* (*Plp1^CreERT^*-negative littermate) mice were treated as depicted in Figure 1A and analyzed at 4 and 6 weeks of age. Dots represent individual samples (animals), horizontal lines indicate means, error bars are depicted as SEM. *p ≤ 0.05, multiple comparisons, two-way ANOVA with *post-hoc* Tukey’s test; there is no significant interaction; individual *p*-values are depicted for *p* > 0.05. The inset in panel **(A)** depicts a representative histogram displaying relative fluorescence intensity (x-axis) vs. event number (y-axis) gated for O4 positivity; overlaid histogram subtraction (GLT-1 histogram—IgG isotype control histogram) was used to determine the percentage of GLT-1-positive maturing (O4-positive) OLGs. **(B)** Representative genomic PCR results for brain tissue taken from *Glt-1 ^Plp^*^1^*icKO* and *Wt* (*Plp1^CreERT^*-negative littermate) female and male mice.

In light of the rather unexpected differences in myelination phenotype seen between males and females (Figure 1), flow cytometry was performed to assess the presence of GLT-1 on maturing OLGs. O4 antibodies were used to mark OLG lineage cells that are in the process of differentiation (Bansal et al., 1989). As shown in Figure 2A, at 4 weeks of age, a significant reduction in the percentage of GLT-1 positive maturing OLGs was observed for male but not female *Glt-1 ^Plp^*^1^*icKO* brains. Based on genomic PCR analysis, no significant differences in recombination efficiency were noted between male and female brain tissues (Figure 2B). Interestingly, it has been reported that there is a higher turnover of OLGs in female brains compared to male brains (Cerghet et al., 2006, 2009), suggesting that in female brains GLT-1 deficient OLGs may have been replaced by newly differentiated OLG progenitor cells that had remained unaffected by the initial 4-HT injections at postnatal days 14, 16, and 17. Indeed, when assessing maturing OLGs at 6 weeks of age, there was no difference in the percentage of GLT-1 positive maturing OLGs in the brains of both male and female *Glt-1 ^Plp^*^1^*icKO* mice (Figure 2C). Thus, in *Glt-1 ^Plp^*^1^*icKO* mice, recombination induced by postnatal 4-HT injections leads to a transient reduction in GLT-1 positive maturing OLGs in male brains that is functionally associated with an attenuation of CNS myelination characterized by decreases in myelination and myelin thickness of predominantly smaller diameter axons. In this context, the slight shift toward smaller diameter myelinated axons seen in the corpus callosum of female *Glt-1 ^Plp^*^1^*icKO* mice (Figure 1C) may be a reflection of a compensatory mechanism in response to a shorter period (compared to males) of reduced percentages of GLT-1 positive maturing OLGs in female brains.

**FIGURE 3:**
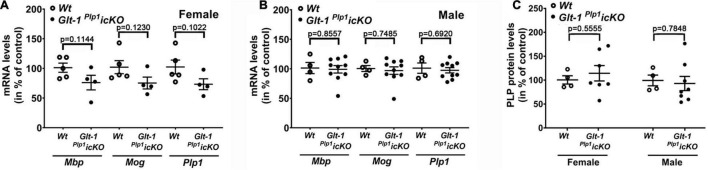
Conditional deletion of *Glt-1* from maturing OLGs does not significantly affect myelin gene expression in the corpus callosum of female and male mice. **(A,B)** Graphs depicting mRNA levels for the myelin genes genes *myelin basic protein (Mbp)*, *myelin oligodendrocyte glycoprotein (Mog)*, and *proteolipid protein (Plp1)* in female **(A)** and male **(B)** corpus callosum as determined by reverse transcriptase quantitative polymerase chain reaction (RT-qPCR). **(C)** Graph depicting PLP protein levels as determined by Western blot analysis. *Glt-1 ^Plp^*^1^*icKO* and *Wt* (*Plp1^CreERT^*-negative littermate) mice were treated as depicted in Figure 1A and analyzed at 4 weeks of age. Dots represent individual samples (animals), horizontal lines indicate means, error bars are depicted as SEM; individual *p*-values are depicted for *p* > 0.05, unpaired *t*-test (two-tailed).

In our previous studies, using cultures of differentiating OLGs, the effects of GLT-1 activation on OLG maturation were selective for morphological aspects and largely unrelated to a regulation of myelin gene expression (Martinez-Lozada et al., 2014; Thomason et al., 2020). Consistent with these *in vitro* findings, no differences in myelin gene expression or proteolipid protein (PLP) levels were noted in the corpus callosum between *Glt-1 ^Plp^*^1^*icKO* mice and *Wt* (*Plp1^CreERT^*-negative littermate) mice (Figure 3). Thus, taken together, our data demonstrate that postnatal loss of GLT-1 in maturing OLGs attenuates myelination in primarily male mice without significantly affecting myelin gene expression.

## Discussion

The data presented in this study identified the sodium-dependent glutamate transporter GLT-1 (EAAT2, SLC1A2) as a novel modulator of developmental CNS myelination. More specifically, our data revealed that deletion of GLT-1 in maturing OLGs during a peak period of developmental myelination attenuates CNS myelination in the corpus callosum of primarily male mice as evidenced by a decreased percentage of smaller diameter myelinated axons and thinner myelin sheaths. These alterations in myelination were not found associated with major changes in myelin gene expression, thus suggesting that mechanisms downstream of GLT-1 activation in maturing OLGs are primarily involved in regulating the morphological aspects of OLG maturation and their association with the initiation of CNS myelination and the thickening of CNS myelin sheaths.

Based on our previous studies (Martinez-Lozada et al., 2014; Spencer et al., 2020; Suarez-Pozos et al., 2020; Thomason et al., 2020), the downstream mechanisms mediating GLT-1’s modulatory effects on CNS myelination are, at least in part, mediated by intracellular calcium transients. In this context, it is notable that calcium transients have been observed in actively myelinating internodes, whereby neuronal activity dependent and independent mechanisms have been described (Micu et al., 2016; Baraban et al., 2018; Krasnow et al., 2018; Battefeld et al., 2019). In addition, localized calcium signals at points of contact between individual OLG processes and axons have been implicated in the initiation of myelination of preferentially electrically active axons by mechanisms that involve non-synaptic vesicular release of glutamate and locally restricted translation of the myelin protein MBP (Wake et al., 2011, 2015). Thus, GLT-1 expressed by maturing OLGs may represent one of the glutamate responsive transmembrane proteins sensing non-synaptic glutamate release by electrically active axons and triggering modulatory changes in CNS myelination. Such modulation of myelination is to be seen as an adaptive mechanism, also referred to as myelin plasticity, that fine-tunes developmental myelination by addition of new myelin and modifications to pre-existing myelin sheaths (Liu et al., 2012; Makinodan et al., 2012; Hill et al., 2018; Hughes et al., 2018; Bacmeister et al., 2020), and by complementing a so-called intrinsic process of myelination, which is thought to occur independently of axonal signals (Lee S. et al., 2012; Bechler et al., 2015, 2018; Mayoral et al., 2018). The concept of adaptive myelination from the initial stages of myelination onward and regulated by the properties of individual axons is further supported by *in vivo* imaging studies done in the developing zebrafish (Hines et al., 2015; Mensch et al., 2015; Koudelka et al., 2016; Nelson et al., 2020). Importantly, the extent and timing of CNS myelination, and thus their modulation by electrical activity, contributes significantly to neuronal circuitry function and its behavioral outputs (Forbes and Gallo, 2017; Bonnefil et al., 2019; Suminaite et al., 2019; Liu et al., 2020; Moore et al., 2020; Pan et al., 2020; Steadman et al., 2020; Xin and Chan, 2020; Fletcher et al., 2021). Notably, in addition to the above signaling downstream of activation of GLT-1, there is the potential of a role of GLT-1 imported glutamate in regulating metabolism. In this context, lipid and protein synthesis during active myelination have been well-established to impose high energetic costs (Harris and Attwell, 2012; Rosko et al., 2019; Tepavcevic, 2021), and an increasing number of studies point toward a high demand of oxidative phosphorylation/mitochondrial metabolism in actively myelinating cells during development. Independent of the exact molecular mechanism, the functional role of GLT-1 in modulating CNS myelination, as described here, coincides with a developmental time window of particular vulnerability for adaptive white matter alterations associated with adult behavioral and cognitive dysfunctions (Kikusui et al., 2007; Liu et al., 2012; Makinodan et al., 2012). In addition, *EAAT2*/*Glt-1* polymorphisms and mutations have been associated with cognitive defects in schizophrenia (Spangaro et al., 2014, 2018; Fiorentino et al., 2015; Mazza et al., 2019) and an increased risk for autism (Autism Genome Project Consortium, Szatmari et al., 2007; Xu et al., 2008), respectively. These findings raise the intriguing possibility that GLT-1 function in maturing OLGs may critically contribute to behaviors regulated by adaptive myelination and that GLT-1 dysfunction in maturing OLGs may represent a critical component of the pathophysiology and behavioral symptoms in certain neuropsychiatric disorders.

## Data Availability Statement

The raw data supporting the conclusions of this article will be made available by the authors, without undue reservation.

## Ethics Statement

All animal studies were approved by the Institutional Animal Care and Use Committees at Virginia Commonwealth University (IACUC #AM10229).

## Author Contributions

ET, ES-P, JD, and BF were directly involved in the design of the study, the analysis of data, and the preparation of the manuscript. FA performed experiments, acquired data, and contributed to method optimization. PR provided study materials and contributed critical revisions to the manuscript. All authors gave consent for publication.

## Conflict of Interest

BF was a consultant for Gryphon Bio, Inc. The remaining authors declare that the research was conducted in the absence of any commercial or financial relationships that could be construed as a potential conflict of interest.

## Publisher’s Note

All claims expressed in this article are solely those of the authors and do not necessarily represent those of their affiliated organizations, or those of the publisher, the editors and the reviewers. Any product that may be evaluated in this article, or claim that may be made by its manufacturer, is not guaranteed or endorsed by the publisher.

## References

[B1] Arranz A. M. Hussein A. Alix J. J. Perez-Cerda F. Allcock N. Matute C. (2008). Functional glutamate transport in rodent optic nerve axons and glia. *Glia* 56 1353–1367. 10.1002/glia.20703 18551624

[B2] Autism Genome Project Consortium, Szatmari P. Paterson A. D. Zwaigenbaum L. Roberts W. Brian J. (2007). Mapping autism risk loci using genetic linkage and chromosomal rearrangements. *Nat. Genet.* 39 319–328. 10.1038/ng1985 17322880 PMC4867008

[B3] Bacmeister C. M. Barr H. J. McClain C. R. Thornton M. A. Nettles D. Welle C. G. (2020). Motor learning promotes remyelination via new and surviving oligodendrocytes. *Nat. Neurosci.* 23 819–831. 10.1038/s41593-020-0637-3 32424285 PMC7329620

[B4] Bansal R. Warrington A. E. Gard A. L. Ranscht B. Pfeiffer S. E. (1989). Multiple and novel specificities of monoclonal antibodies O1, O4, and R-mAb used in the analysis of oligodendrocyte development. *J. Neurosci. Res.* 24 548–557. 10.1002/jnr.490240413 2600978

[B5] Baraban M. Koudelka S. Lyons D. A. (2018). Ca (2+) activity signatures of myelin sheath formation and growth in vivo. *Nat. Neurosci.* 21 19–23. 10.1038/s41593-017-0040-x 29230058 PMC5742537

[B6] Battefeld A. Popovic M. A. de Vries S. I. Kole M. H. P. (2019). High-Frequency Microdomain Ca(2+) Transients and Waves during Early Myelin Internode Remodeling. *Cell Rep.* 26 182–191.e5. 10.1016/j.celrep.2018.12.039 30605675 PMC6316190

[B7] Bechler M. E. Byrne L. Ffrench-Constant C. (2015). CNS Myelin Sheath Lengths Are an Intrinsic Property of Oligodendrocytes. *Curr. Biol.* 25 2411–2416. 10.1016/j.cub.2015.07.056 26320951 PMC4580335

[B8] Bechler M. E. Swire M. Ffrench-Constant C. (2018). Intrinsic and adaptive myelination-A sequential mechanism for smart wiring in the brain. *Dev. Neurobiol.* 78 68–79. 10.1002/dneu.22518 28834358 PMC5813148

[B9] Benusa S. D. George N. M. Sword B. A. DeVries G. H. Dupree J. L. (2017). Acute neuroinflammation induces AIS structural plasticity in a NOX2-dependent manner. *J. Neuroinflammation* 14:116. 10.1186/s12974-017-0889-3 28595650 PMC5465457

[B10] Bin J. M. Harris S. N. Kennedy T. E. (2016). The oligodendrocyte-specific antibody ‘CC1’ binds Quaking 7. *J. Neurochem.* 139 181–186. 10.1111/jnc.13745 27454326

[B11] Bonnefil V. Dietz K. Amatruda M. Wentling M. Aubry A. V. Dupree J. L. (2019). Region-specific myelin differences define behavioral consequences of chronic social defeat stress in mice. *Elife* 8:e40855. 10.7554/eLife.40855 31407664 PMC6692108

[B12] Bustin S. A. Benes V. Garson J. A. Hellemans J. Huggett J. Kubista M. (2009). The MIQE guidelines: minimum information for publication of quantitative real-time PCR experiments. *Clin. Chem.* 55 611–622. 10.1373/clinchem.2008.112797 19246619

[B13] Caley D. W. Maxwell D. S. (1968). An electron microscopic study of neurons during postnatal development of the rat cerebral cortex. *J. Comp. Neurol.* 133 17–44. 10.1002/cne.901330103 5721481

[B14] Cerghet M. Skoff R. P. Bessert D. Zhang Z. Mullins C. Ghandour M. S. (2006). Proliferation and death of oligodendrocytes and myelin proteins are differentially regulated in male and female rodents. *J. Neurosci.* 26 1439–1447. 10.1523/JNEUROSCI.2219-05.2006 16452667 PMC6675481

[B15] Cerghet M. Skoff R. P. Swamydas M. Bessert D. (2009). Sexual dimorphism in the white matter of rodents. *J. Neurol. Sci.* 286 76–80. 10.1016/j.jns.2009.06.039 19625027 PMC2760672

[B16] Chen C. J. Ou Y. C. Lin S. Y. Liao S. L. Huang Y. S. Chiang A. N. (2006). L-glutamate activates RhoA GTPase leading to suppression of astrocyte stellation. *Eur. J. Neurosci.* 23 1977–1987. 10.1111/j.1460-9568.2006.04728.x 16630046

[B17] Dalgaard P. (2008). *Introductory Statistics with R.* New York: Springer.

[B18] Danbolt N. C. (2001). Glutamate uptake. *Prog. Neurobiol.* 65 1–105. 10.1016/s0301-0082(00)00067-8 11369436

[B19] DeSilva T. M. Kabakov A. Y. Goldhoff P. E. Volpe J. J. Rosenberg P. A. (2009). Regulation of glutamate transport in developing rat oligodendrocytes. *J. Neurosci.* 29 7898–7908. 10.1523/JNEUROSCI.6129-08.2009 19535601 PMC2926807

[B20] Doerflinger N. H. Macklin W. B. Popko B. (2003). Inducible site-specific recombination in myelinating cells. *Genesis* 35 63–72. 10.1002/gene.10154 12481300

[B21] Domercq M. Matute C. (1999). Expression of glutamate transporters in the adult bovine corpus callosum. *Brain Res. Mol. Brain Res.* 67 296–302. 10.1016/s0169-328x(99)00072-8 10216228

[B22] Dupree J. L. Coetzee T. Suzuki K. Popko B. (1998). Myelin abnormalities in mice deficient in galactocerebroside and sulfatide. *J. Neurocytol.* 27 649–659. 10.1023/a:1006908013972 10447239

[B23] Dupree J. L. Girault J. A. Popko B. (1999). Axo-glial interactions regulate the localization of axonal paranodal proteins. *J. Cell Biol.* 147 1145–1152. 10.1083/jcb.147.6.1145 10601330 PMC2168103

[B24] Fiorentino A. Sharp S. I. McQuillin A. (2015). Association of rare variation in the glutamate receptor gene SLC1A2 with susceptibility to bipolar disorder and schizophrenia. *Eur. J. Hum. Genet.* 23 1200–1206. 10.1038/ejhg.2014.261 25406999 PMC4351899

[B25] Fletcher J. L. Makowiecki K. Cullen C. L. Young K. M. (2021). Oligodendrogenesis and myelination regulate cortical development, plasticity and circuit function. *Semin. Cell Dev. Biol.* 118 14–23. 10.1016/j.semcdb.2021.03.017 33863642

[B26] Flores-Mendez M. A. Martinez-Lozada Z. Monroy H. C. Hernandez-Kelly L. C. Barrera I. Ortega A. (2013). Glutamate-dependent translational control in cultured Bergmann glia cells: eIF2alpha phosphorylation. *Neurochem. Res.* 38 1324–1332. 10.1007/s11064-013-1024-1 23536022

[B27] Foran D. R. Peterson A. C. (1992). Myelin acquisition in the central nervous system of the mouse revealed by an MBP-Lac Z transgene. *J. Neurosci.* 12 4890–4897. 10.1523/JNEUROSCI.12-12-04890.1992 1281497 PMC6575777

[B28] Forbes T. A. Gallo V. (2017). All Wrapped Up: environmental Effects on Myelination. *Trends Neurosci.* 40 572–587. 10.1016/j.tins.2017.06.009 28844283 PMC5671205

[B29] Forrest A. D. Beggs H. E. Reichardt L. F. Dupree J. L. Colello R. J. Fuss B. (2009). Focal adhesion kinase (FAK): a regulator of CNS myelination. *J. Neurosci. Res.* 87 3456–3464. 10.1002/jnr.22022 19224576 PMC2760606

[B30] Fuss B. Mallon B. Phan T. Ohlemeyer C. Kirchhoff F. Nishiyama A. (2000). Purification and analysis of in vivo-differentiated oligodendrocytes expressing the green fluorescent protein. *Dev. Biol.* 218 259–274. 10.1006/dbio.1999.9574 10656768

[B31] Guo F. Ma J. McCauley E. Bannerman P. Pleasure D. (2009). Early postnatal proteolipid promoter-expressing progenitors produce multilineage cells in vivo. *J. Neurosci.* 29 7256–7270. 10.1523/JNEUROSCI.5653-08.2009 19494148 PMC2717630

[B32] Harris J. J. Attwell D. (2012). The energetics of CNS white matter. *J. Neurosci.* 32 356–371. 10.1523/JNEUROSCI.3430-11.2012 22219296 PMC3272449

[B33] Hill R. A. Li A. M. Grutzendler J. (2018). Lifelong cortical myelin plasticity and age-related degeneration in the live mammalian brain. *Nat. Neurosci.* 21 683–695. 10.1038/s41593-018-0120-6 29556031 PMC5920745

[B34] Hines J. H. Ravanelli A. M. Schwindt R. Scott E. K. Appel B. (2015). Neuronal activity biases axon selection for myelination in vivo. *Nat. Neurosci.* 18 683–689. 10.1038/nn.3992 25849987 PMC4414883

[B35] Hughes E. G. Orthmann-Murphy J. L. Langseth A. J. Bergles D. E. (2018). Myelin remodeling through experience-dependent oligodendrogenesis in the adult somatosensory cortex. *Nat. Neurosci.* 21 696–706. 10.1038/s41593-018-0121-5 29556025 PMC5920726

[B36] Jeffries M. A. Urbanek K. Torres L. Wendell S. G. Rubio M. E. Fyffe-Maricich S. L. (2016). ERK1/2 Activation in Preexisting Oligodendrocytes of Adult Mice Drives New Myelin Synthesis and Enhanced CNS Function. *J. Neurosci.* 36 9186–9200. 10.1523/JNEUROSCI.1444-16.2016 27581459 PMC5005725

[B37] Kang S. H. Fukaya M. Yang J. K. Rothstein J. D. Bergles D. E. (2010). NG2+ CNS glial progenitors remain committed to the oligodendrocyte lineage in postnatal life and following neurodegeneration. *Neuron* 68 668–681. 10.1016/j.neuron.2010.09.009 21092857 PMC2989827

[B38] Karlsson U. Schultz R. L. (1965). Fixation of the Central Nervous System from Electron Microscopy by Aldehyde Perfusion. I. Preservation with Aldehyde Perfusates Versus Direct Perfusion with Osmium Tetroxide with Special Reference to Membranes and the Extracellular Space. *J. Ultrastruct. Res.* 12 160–186. 10.1016/s0022-5320(65)80014-4 14289426

[B39] Kikusui T. Kiyokawa Y. Mori Y. (2007). Deprivation of mother-pup interaction by early weaning alters myelin formation in male, but not female ICR mice. *Brain Res.* 1133 115–122. 10.1016/j.brainres.2006.11.031 17184748

[B40] Koenning M. Jackson S. Hay C. M. Faux C. Kilpatrick T. J. Willingham M. (2012). Myelin gene regulatory factor is required for maintenance of myelin and mature oligodendrocyte identity in the adult CNS. *J. Neurosci.* 32 12528–12542. 10.1523/JNEUROSCI.1069-12.2012 22956843 PMC3752083

[B41] Koudelka S. Voas M. G. Almeida R. G. Baraban M. Soetaert J. Meyer M. P. (2016). Individual Neuronal Subtypes Exhibit Diversity in CNS Myelination Mediated by Synaptic Vesicle Release. *Curr. Biol.* 26 1447–1455. 10.1016/j.cub.2016.03.070 27161502 PMC4906267

[B42] Krasnow A. M. Ford M. C. Valdivia L. E. Wilson S. W. Attwell D. (2018). Regulation of developing myelin sheath elongation by oligodendrocyte calcium transients in vivo. *Nat. Neurosci.* 21 24–28. 10.1038/s41593-017-0031-y 29230052 PMC6478117

[B43] Kukley M. Nishiyama A. Dietrich D. (2010). The fate of synaptic input to NG2 glial cells: neurons specifically downregulate transmitter release onto differentiating oligodendroglial cells. *J. Neurosci.* 30 8320–8331. 10.1523/JNEUROSCI.0854-10.2010 20554883 PMC6634580

[B44] Laprairie R. B. Petr G. T. Sun Y. Fischer K. D. Denovan-Wright E. M. Rosenberg P. A. (2019). Huntington’s disease pattern of transcriptional dysregulation in the absence of mutant huntingtin is produced by knockout of neuronal GLT-1. *Neurochem. Int.* 123 85–94. 10.1016/j.neuint.2018.04.015 29709465 PMC6249114

[B45] Lee A. Anderson A. R. Beasley S. J. Barnett N. L. Poronnik P. Pow D. V. (2012). A new splice variant of the glutamate-aspartate transporter: cloning and immunolocalization of GLAST1c in rat, pig and human brains. *J. Chem. Neuroanat.* 43 52–63. 10.1016/j.jchemneu.2011.10.005 22026960

[B46] Lee S. Leach M. K. Redmond S. A. Chong S. Y. Mellon S. H. Tuck S. J. (2012). A culture system to study oligodendrocyte myelination processes using engineered nanofibers. *Nat. Methods* 9 917–922. 10.1038/nmeth.2105 22796663 PMC3433633

[B47] Leone D. P. Genoud S. Atanasoski S. Grausenburger R. Berger P. Metzger D. (2003). Tamoxifen-inducible glia-specific Cre mice for somatic mutagenesis in oligodendrocytes and Schwann cells. *Mol. Cell Neurosci.* 22 430–440. 10.1016/s1044-7431(03)00029-0 12727441

[B48] Liu J. Dietz K. DeLoyht J. M. Pedre X. Kelkar D. Kaur J. (2012). Impaired adult myelination in the prefrontal cortex of socially isolated mice. *Nat. Neurosci.* 15 1621–1623. 10.1038/nn.3263 23143512 PMC3729624

[B49] Liu J. Likhtik E. Shereen A. D. Dennis-Tiwary T. A. Casaccia P. (2020). White Matter Plasticity in Anxiety: disruption of Neural Network Synchronization During Threat-Safety Discrimination. *Front. Cell Neurosci.* 14:587053. 10.3389/fncel.2020.587053 33250713 PMC7674975

[B50] Livak K. J. Schmittgen T. D. (2001). Analysis of relative gene expression data using real-time quantitative PCR and the 2(-Delta Delta C(T)) Method. *Methods* 25 402–408. 10.1006/meth.2001.1262 11846609

[B51] Makinodan M. Rosen K. M. Ito S. Corfas G. (2012). A critical period for social experience-dependent oligodendrocyte maturation and myelination. *Science* 337 1357–1360. 10.1126/science.1220845 22984073 PMC4165613

[B52] Marcus J. Honigbaum S. Shroff S. Honke K. Rosenbluth J. Dupree J. L. (2006). Sulfatide is essential for the maintenance of CNS myelin and axon structure. *Glia* 53 372–381. 10.1002/glia.20292 16288467

[B53] Maria Lopez-Colome A. Martinez-Lozada Z. Guillem A. M. Lopez E. Ortega A. (2012). Glutamate transporter-dependent mTOR phosphorylation in Muller glia cells. *ASN Neuro* 4:e00095. 10.1042/AN20120022 22817638 PMC3420017

[B54] Martinez-Lozada Z. Hernandez-Kelly L. C. Aguilera J. Lopez-Bayghen E. Ortega A. (2011). Signaling through EAAT-1/GLAST in cultured Bergmann glia cells. *Neurochem* 59 871–879. 10.1016/j.neuint.2011.07.015 21856347

[B55] Martinez-Lozada Z. Waggener C. T. Kim K. Zou S. Knapp P. E. Hayashi Y. (2014). Activation of sodium-dependent glutamate transporters regulates the morphological aspects of oligodendrocyte maturation via signaling through calcium/calmodulin-dependent kinase IIbeta’s actin-binding/-stabilizing domain. *Glia* 62 1543–1558. 10.1002/glia.22699 24866099 PMC4107011

[B56] Mason J. L. Langaman C. Morell P. Suzuki K. Matsushima G. K. (2001). Episodic demyelination and subsequent remyelination within the murine central nervous system: changes in axonal calibre. *Neuropathol. Appl. Neurobiol.* 27 50–58. 10.1046/j.0305-1846.2001.00301.x 11299002

[B57] Mayoral S. R. Etxeberria A. Shen Y. A. Chan J. R. (2018). Initiation of CNS Myelination in the Optic Nerve Is Dependent on Axon Caliber. *Cell Rep.* 25 544-550.e3. 10.1016/j.celrep.2018.09.052 30332636 PMC6258034

[B58] Mazza E. Spangaro M. Poletti S. Cavallaro R. Benedetti F. (2019). Genetic variability of glutamate reuptake: effect on white matter integrity and working memory in schizophrenia. *Schizophr. Res.* 208 457–459. 10.1016/j.schres.2019.03.004 30857874

[B59] McNair L. F. Andersen J. V. Aldana B. I. Hohnholt M. C. Nissen J. D. Sun Y. (2019). Deletion of Neuronal GLT-1 in Mice Reveals Its Role in Synaptic Glutamate Homeostasis and Mitochondrial Function. *J. Neurosci.* 39 4847–4863. 10.1523/JNEUROSCI.0894-18.2019 30926746 PMC6670249

[B60] McNair L. F. Andersen J. V. Nissen J. D. Sun Y. Fischer K. D. Hodgson N. W. (2020). Conditional Knockout of GLT-1 in Neurons Leads to Alterations in Aspartate Homeostasis and Synaptic Mitochondrial Metabolism in Striatum and Hippocampus. *Neurochem. Res.* 45 1420–1437. 10.1007/s11064-020-03000-7 32144526

[B61] Mensch S. Baraban M. Almeida R. Czopka T. Ausborn J. El Manira A. (2015). Synaptic vesicle release regulates myelin sheath number of individual oligodendrocytes in vivo. *Nat. Neurosci.* 18 628–630. 10.1038/nn.3991 25849985 PMC4427868

[B62] Michalski J. P. Anderson C. Beauvais A. De Repentigny Y. Kothary R. (2011). The proteolipid protein promoter drives expression outside of the oligodendrocyte lineage during embryonic and early postnatal development. *PLoS One* 6:e19772. 10.1371/journal.pone.0019772 21572962 PMC3091881

[B63] Micu I. Plemel J. R. Lachance C. Proft J. Jansen A. J. Cummins K. (2016). The molecular physiology of the axo-myelinic synapse. *Exp. Neurol.* 276 41–50. 10.1016/j.expneurol.2015.10.006 26515690

[B64] Moore S. Meschkat M. Ruhwedel T. Trevisiol A. Tzvetanova I. D. Battefeld A. (2020). A role of oligodendrocytes in information processing. *Nat. Commun.* 11:5497. 10.1038/s41467-020-19152-7 33127910 PMC7599337

[B65] Nelson H. N. Treichel A. J. Eggum E. N. Martell M. R. Kaiser A. J. Trudel A. G. (2020). Individual neuronal subtypes control initial myelin sheath growth and stabilization. *Neural Dev.* 15:12. 10.1186/s13064-020-00149-3 32988384 PMC7523326

[B66] Pan S. Mayoral S. R. Choi H. S. Chan J. R. Kheirbek M. A. (2020). Preservation of a remote fear memory requires new myelin formation. *Nat. Neurosci.* 23 487–499. 10.1038/s41593-019-0582-1 32042175 PMC7213814

[B67] Petr G. T. Sun Y. Frederick N. M. Zhou Y. Dhamne S. C. Hameed M. Q. (2015). Conditional deletion of the glutamate transporter GLT-1 reveals that astrocytic GLT-1 protects against fatal epilepsy while neuronal GLT-1 contributes significantly to glutamate uptake into synaptosomes. *J. Neurosci.* 35 5187–5201. 10.1523/JNEUROSCI.4255-14.2015 25834045 PMC4380995

[B68] Regan M. R. Huang Y. H. Kim Y. S. Dykes-Hoberg M. I. Jin L. Watkins A. M. (2007). Variations in promoter activity reveal a differential expression and physiology of glutamate transporters by glia in the developing and mature CNS. *J. Neurosci.* 27 6607–6619. 10.1523/JNEUROSCI.0790-07.2007 17581948 PMC6672708

[B69] Rimmele T. S. Rosenberg P. A. (2016). GLT-1: the elusive presynaptic glutamate transporter. *Neurochem. Int.* 98 19–28. 10.1016/j.neuint.2016.04.010 27129805 PMC5070539

[B70] Rimmele T. S. Li S. Andersen J. V. Westi E. W. Rotenberg A. Wang J. (2021). Neuronal Loss of the Glutamate Transporter GLT-1 Promotes Excitotoxic Injury in the Hippocampus. *Front. Cell Neurosci.* 15:788262. 10.3389/fncel.2021.788262 35035352 PMC8752461

[B71] Robinson M. B. Jackson J. G. (2016). Astroglial glutamate transporters coordinate excitatory signaling and brain energetics. *Neurochem. Int.* 98 56–71. 10.1016/j.neuint.2016.03.014 27013346 PMC4969184

[B72] Robinson M. B. Lee M. L. DaSilva S. (2020). Glutamate Transporters and Mitochondria: signaling, Co-compartmentalization, Functional Coupling, and Future Directions. *Neurochem. Res.* 45 526–540. 10.1007/s11064-020-02974-8 32002773 PMC7060825

[B73] Rose C. R. Ziemens D. Untiet V. Fahlke C. (2018). Molecular and cellular physiology of sodium-dependent glutamate transporters. *Brain Res. Bull.* 136 3–16. 10.1016/j.brainresbull.2016.12.013 28040508

[B74] Rosko L. Smith V. N. Yamazaki R. Huang J. K. (2019). Oligodendrocyte Bioenergetics in Health and Disease. *Neuroscientist* 25 334–343. 10.1177/1073858418793077 30122106 PMC6745601

[B75] Rothstein J. D. Dykes-Hoberg M. Pardo C. A. Bristol L. A. Jin L. Kuncl R. W. (1996). Knockout of glutamate transporters reveals a major role for astroglial transport in excitotoxicity and clearance of glutamate. *Neuron* 16 675–686. 10.1016/s0896-6273(00)80086-0 8785064

[B76] Schindelin J. Arganda-Carreras I. Frise E. Kaynig V. Longair M. Pietzsch T. (2012). Fiji: an open-source platform for biological-image analysis. *Nat. Methods* 9 676–682. 10.1038/nmeth.2019 22743772 PMC3855844

[B77] Seixas A. I. Azevedo M. M. Paes de Faria J. Fernandes D. Mendes Pinto I. Relvas J. B. (2019). Evolvability of the actin cytoskeleton in oligodendrocytes during central nervous system development and aging. *Cell Mol. Life Sci.* 76 1–11. 10.1007/s00018-018-2915-8 30302529 PMC11105620

[B78] Skokal R. R. Rohlf F. J. (1995). *Biometry: The Principle and Practice in Biological Research.* New York: W. H. Freeman and Company.

[B79] Spangaro M. Bosia M. Bechi M. Buonocore M. Cocchi F. Guglielmino C. (2018). Neurobiology of cognitive remediation in schizophrenia: effects of EAAT2 polymorphism. *Schizophr. Res.* 202 106–110. 10.1016/j.schres.2018.06.059 30539765

[B80] Spangaro M. Bosia M. Zanoletti A. Bechi M. Mariachiara B. Pirovano A. (2014). Exploring effects of EAAT polymorphisms on cognitive functions in schizophrenia. *Pharmacogenomics* 15 925–932. 10.2217/pgs.14.42 24956246

[B81] Spencer S. A. Suarez-Pozos E. Escalante M. Myo Y. P. Fuss B. (2020). Sodium-Calcium Exchangers of the SLC8 Family in Oligodendrocytes: functional Properties in Health and Disease. *Neurochem. Res.* 45 1287–1297. 10.1007/s11064-019-02949-4 31927687 PMC7885042

[B82] Srinivas S. Watanabe T. Lin C. S. William C. M. Tanabe Y. Jessell T. M. (2001). Cre reporter strains produced by targeted insertion of EYFP and ECFP into the ROSA26 locus. *BMC Dev. Biol.* 1:4. 10.1186/1471-213x-1-4 11299042 PMC31338

[B83] Steadman P. E. Xia F. Ahmed M. Mocle A. J. Penning A. R. A. Geraghty A. C. (2020). Disruption of Oligodendrogenesis Impairs Memory Consolidation in Adult Mice. *Neuron* 105 150–164.e6. 10.1016/j.neuron.2019.10.013 31753579 PMC7579726

[B84] Suarez-Pozos E. Thomason E. J. Fuss B. (2020). Glutamate Transporters: expression and Function in Oligodendrocytes. *Neurochem. Res.* 45 551–560. 10.1007/s11064-018-02708-x 30628017 PMC6616022

[B85] Suminaite D. Lyons D. A. Livesey M. R. (2019). Myelinated axon physiology and regulation of neural circuit function. *Glia* 67 2050–2062. 10.1002/glia.23665 31233642 PMC6772175

[B86] Tanaka K. Watase K. Manabe T. Yamada K. Watanabe M. Takahashi K. (1997). Epilepsy and exacerbation of brain injury in mice lacking the glutamate transporter GLT-1. *Science* 276 1699–1702. 10.1126/science.276.5319.1699 9180080

[B87] Tepavcevic V. (2021). Oligodendroglial Energy Metabolism and (re)Myelination. *Life* 11 238. 10.3390/life11030238 33805670 PMC7998845

[B88] Thomason E. J. Escalante M. Osterhout D. J. Fuss B. (2020). The oligodendrocyte growth cone and its actin cytoskeleton: a fundamental element for progenitor cell migration and CNS myelination. *Glia* 68 1329–1346. 10.1002/glia.23735 31696982 PMC7942813

[B89] Traka M. Arasi K. Avila R. L. Podojil J. R. Christakos A. Miller S. D. (2010). A genetic mouse model of adult-onset, pervasive central nervous system demyelination with robust remyelination. *Brain* 133 3017–3029. 10.1093/brain/awq247 20851998 PMC4415057

[B90] Waggener C. T. Dupree J. L. Elgersma Y. Fuss B. (2013). CaMKIIbeta regulates oligodendrocyte maturation and CNS myelination. *J. Neurosci.* 33 10453–10458. 10.1523/JNEUROSCI.5875-12.2013 23785157 PMC3685839

[B91] Wake H. Lee P. R. Fields R. D. (2011). Control of local protein synthesis and initial events in myelination by action potentials. *Science* 333 1647–1651. 10.1126/science.1206998 21817014 PMC3482340

[B92] Wake H. Ortiz F. C. Woo D. H. Lee P. R. Angulo M. C. Fields R. D. (2015). Nonsynaptic junctions on myelinating glia promote preferential myelination of electrically active axons. *Nat. Commun.* 6:7844. 10.1038/ncomms8844 26238238 PMC4532789

[B93] Xin W. Chan J. R. (2020). Myelin plasticity: sculpting circuits in learning and memory. *Nat. Rev. Neurosci.* 21 682–694. 10.1038/s41583-020-00379-8 33046886 PMC8018611

[B94] Xu S. Han J. C. Morales A. Menzie C. M. Williams K. Fan Y. S. (2008). Characterization of 11p14-p12 deletion in WAGR syndrome by array CGH for identifying genes contributing to mental retardation and autism. *Cytogenet. Genome Res.* 122 181–187. 10.1159/000172086 19096215

[B95] Yamamura T. Konola J. T. Wekerle H. Lees M. B. (1991). Monoclonal antibodies against myelin proteolipid protein: identification and characterization of two major determinants. *J. Neurochem.* 57 1671–1680. 10.1111/j.1471-4159.1991.tb06367.x 1717653

[B96] Ye J. Coulouris G. Zaretskaya I. Cutcutache I. Rozen S. Madden T. L. (2012). Primer-BLAST: a tool to design target-specific primers for polymerase chain reaction. *BMC Bioinform.* 13:134. 10.1186/1471-2105-13-134 22708584 PMC3412702

[B97] Zhou Y. Hassel B. Eid T. Danbolt N. C. (2019). Axon-terminals expressing EAAT2 (GLT-1; Slc1a2) are common in the forebrain and not limited to the hippocampus. *Neurochem. Int.* 123 101–113. 10.1016/j.neuint.2018.03.006 29530756

[B98] Zuchero J. B. Fu M. M. Sloan S. A. Ibrahim A. Olson A. Zaremba A. (2015). CNS myelin wrapping is driven by actin disassembly. *Dev. Cell* 34 152–167. 10.1016/j.devcel.2015.06.011 26166300 PMC4519368

